# Seeing Effort: Assessing Coaches' Prediction of the Number of Repetitions in Reserve Before Task-Failure

**DOI:** 10.1186/s40798-022-00526-8

**Published:** 2022-10-22

**Authors:** Aviv Emanuel, Itai Har-Nir, Uri Obolski, Israel Halperin

**Affiliations:** 1 Department of Health Promotion, School of Public Health, Sackler Faculty of Medicine, Tel-Aviv University, Tel-Aviv, Israel; 2 Sylvan Adams Sports Institute, Tel Aviv University, Tel-Aviv, Israel; 3 School of Psychological Sciences, Tel-Aviv University, Tel-Aviv, Israel; 4 Department of Epidemiology and Preventive Medicine, School of Public Health, Sackler Faculty of Medicine, Tel Aviv University, Tel Aviv, Israel; 5 Department of Environmental Studies, Porter School of the Environment and Earth Sciences, Sackler Faculty of Exact Sciences, Tel Aviv University, Tel Aviv, Israel

**Keywords:** Resistance-training, Repetitions in reserve, Task-failure, Personal training, Coach’s eye

## Abstract

**Background:**

A key role of resistance training (RT) coaches is to personalize programs based on their trainees’ abilities and goals. Specifically, coaches often assess how many repetitions in reserve (RIR) their trainees have until task-failure. Coaches can then modify the number of repetitions assigned per set accordingly. However, coaches’ ability to predict the number of RIR is unknown.

**Methods:**

Certified RT coaches (*n* = 259) were randomly assigned to watch a video of one of eight trainees. The trainees performed two sets of barbell squats and preacher biceps-curls, using 70% or 80% of their 1RM, to task-failure. The coaches predicted trainees’ RIR at 33%, 66%, and 90% of the total number of repetitions the trainees completed in each set. We fitted a linear mixed model with various predictors to the prediction errors as the outcomes (i.e., signed and unsigned values of the predicted minus actual repetitions to task-failure).

**Results:**

The overall average number of repetitions completed by the trainees was 13.9. The average absolute errors were 4.8, 2.0, and 1.2 repetitions for the 33%, 66%, and 90% time-points, respectively. The absolute prediction error increased for the biceps-curl compared to the squat (1.43, 95% CI [1.13, 1.74]), but decreased for heavier loads (− 1.17, 95% CI [− 2.16, − 0.19]), and in the second set of each exercise (− 1.20, 95% CI [− 1.38, − 1.02]). Surprisingly, coaches’ years of experience had a negligible effect on the absolute error (− 0.020, 95% CI [− 0.039, − 0.0007]). Finally, coaches underpredicted the RIR at early time-points but reverted to slight overprediction at later time-points.

**Conclusions:**

Prior coaching experience seems to play a minor role in RIR predictions. However, even short-term exposures to new trainees performing different exercises can substantially improve coaches’ RIR predictions.

**Supplementary Information:**

The online version contains supplementary material available at 10.1186/s40798-022-00526-8.

## Key Points

- It is unknown how well coaches can estimate the number of repetitions left before their trainees reach task-failure in resistance exercises.
- We had 259 coaches watch videos of trainees performing two exercises with one of two loads and estimate the number of repetitions left to task-failure at different stages of the sets.
- Exercise type, load, set number, and proximity to task-failure substantially affected the accuracy of coaches’ estimations, whereas experience had a negligible effect.
- Coaches’ ability to predict task-failure tended to improve as the sets progressed, with consecutive sets, and for sets composed of heavier loads.

## Background

Prescribing resistance-training (RT) programs is a complex task. It requires coaches to personalize variables such as exercises, loads, number of repetitions, and sets. Various predetermined RT programs have been developed over the years, targeting specific populations and outcomes [1, 2]. These supply coaches with general RT outlines, simplifying the prescription processes. For example, a RT program composed of 1–3 sets, 8–12 repetitions, and 60–70% of one repetition maximum (1RM) can be prescribed to improve the strength levels of novice and intermediate trainees [1]. RT programs can be further personalized by modifying training variables based on real-time data [3]. This can be achieved by employing questionnaires, tracking bar velocity, or relying on coaches’ observations (i.e., “coach’ eye”) [3]. The coach’s eye can be defined as the coach’s ability to monitor the trainee’s exercise performance for its technical execution and intensity of effort (i.e., distance from task-failure). Notably, despite the growing number of programs, questionnaires, and technological tools aimed to assist in RT prescription, the “coach’s eye” is still considered an essential factor in successful coaching [4, 5].

A prominent RT variable subject to real-time modification is the number of repetitions to be completed per set. The maximal number of repetitions performed for a given exercise while lifting a certain percentage of 1RM varies significantly within (and between) individuals. For example, the maximal number of repetitions trainees can complete is affected by mental fatigue [6], whether they ingested caffeine [7], their object of focus when exercising [8], and even if their preferred music is played in the background [9]. Due to such expected variance in training conditions, prescribing the same number of repetitions on different days may result in inconsistent levels of intensity of effort. Subsequently, this may lead to inconsistent physiological and psychological responses. Given this variance, the coach’s ability to accurately estimate the intensity of effort exerted in an ongoing set is an essential coaching skill. For example, a coach may notice signs of fatigue during an ongoing set based on the trainee’s facial expressions, movement velocity, technique execution, and more. Consequently, a coach may instruct the trainee to terminate the set earlier than planned or modify the loads or repetitions in subsequent sets. This process can better align the desired intensity of the RT sessions with the goals of the program.

Despite the importance assigned to the “coach’s eye” in RT [4, 5], the accuracy with which coaches predict trainees’ repetitions in reserve (RIR) before reaching task-failure in an ongoing set has never been studied. In this context, all research concerning RIR has focused on trainees rather than on coaches [10]. In such studies, trainees are instructed to verbally predict the RIR before or during a set, and their prediction accuracy is examined. If sufficient prediction accuracy is reached, trainees can use their prediction of RIR to modify their number of repetitions in real time [11, 12]. By doing so, trainees can better account for the variability in their performance and exercise in a more personalized manner. We propose that it is also of interest to conduct similar study designs that examine coaches’ predictions of trainees’ RIR.

In view of the above, the goal of this study was to assess coaches’ accuracy in predicting trainees’ RIR. To this end, we recruited RT coaches to participate in an online survey. We presented them with videos of one of eight resistance-trained trainees performing two sets, of two exercises, with two loads. At various time-points during the sets, the coaches predicted the trainees’ RIR. We examined whether the following variables influenced prediction accuracy: coaching experience, the timing of prediction, exercises, set number, loads, and trainee.

## Methods

### Participants

We recruited participants by (1) contacting and asking the accredited RT coaching schools in Israel to distribute our survey link to their alumni and (2) posting our survey link on various Facebook groups that focus on personal training and RT. The final sample included 259 RT coaches who provided at least 11 of the 12 RIR predictions. Due to technical errors in the survey platform, complete demographic data were available for only 153 participants (Table 1). A table comparing the characteristics of participants with and without missing data is available as a supplemental file at https://osf.io/fgycv/. The Institution's Ethics Committee approved all procedures.

**Table 1:** Coach characteristics (mean ± SD) (*n* = 259)

Age	29.8 ± 7.5
Weight (kg)	74.1 ± 12.7
Height (cm)	174 ± 0.1
Average workouts per week	4.8 ± 3.32
Gender*	46F and 107M
Hours of RT coaching per week (average)*	14.8 ± 12.6
Years of experience in RT*	9.30 ± 5.8

*Data available for 153 participants

### Procedures

Participants joined the survey by clicking a link sent via email. The link directed them to the Qualtrics platform (Qualtrics XM Platform, Utah, USA), in which they read and electronically signed an informed consent form. Participants were then asked whether they were certified RT coaches via one of the accredited schools in Israel ("*yes/no*"). Note that a RT coaching certificate in Israel consists of a yearlong course of ~ 350 h. In case of a negative response, participants were thanked for their response and notified that the survey had ended. In case of a positive response, participants were directed to a different online platform (www.hapyak.com), where they first answered a series of demographic questions (Table 1). Participants were then presented with the following instructions:

*You will now watch a video of a trainee performing two sets of the squat exercise and two sets of a biceps-curl exercise using 70% or 80% of the maximal load they can lift once (1RM) to task-failure. Task-failure is defined as an event in which the trainee terminates the set because s/he cannot complete another repetition or because s/he estimates to be unable to complete another repetition. Please note that the trainees in the videos have experience in resistance training; they performed all the sets on the same day, rested for about eight minutes between each set, and were to perform the concentric portion of each repetition as fast as possible while attempting to maintain a controlled ~ 2 s descend. When watching the videos, you will be asked to evaluate several times how many repetitions are left before the trainee reaches task-failure. In your answer, please type the digit itself (for example, 3 and not three).*

Subsequently, each coach watched a video of a single trainee performing two sets to task-failure in the barbell squat, followed by two sets to task-failure in the biceps-curl, using either 70% or 80% of their 1RM. Each coach was randomly assigned to watch one of 15 possible videos (eight trainees completed two load conditions on separate days, and one of the 70%1RM videos was corrupted and hence excluded). The videos stopped at 33%, 66%, and 90% of the total repetitions completed in each set during which a box appeared with the following question: “how many repetitions are left before the trainee reaches task-failure?”. Coaches were required to insert a single number before the video continued. Importantly, coaches were oblivious to how many times and when relative to task-failure they were required to provide their predictions.

### Trainees

The RT coaches were randomly assigned to watch a video of one out of eight trainees, all of whom had experience in RT (Table 2). The trainees participated in three sessions: A 1RM testing session (see [13, 14] for more details) in the squat and biceps-curl exercises and two sessions composed of two sets of squats followed by two sets of biceps-curls to task-failure using either 70% or 80% of 1RM, performed on separate days and in a counterbalanced order. We note that only a single 1RM session was conducted as the test–retest reliability of 1RM loads is very high across populations and exercises [15]. The squats were performed within a squat cage and the biceps-curls on a preacher chair. All sessions were conducted in the same facility and supervised by the same experimenter at approximately the same hour of the day (± 2 h). A minimum of three and a maximum of eight days between sessions were allowed. Trainees were asked to refrain from an intense training session 24 h before testing days that may lead to performance decrements and muscle soreness involving the squat and biceps-curls. Trainees were also asked to avoid heavy meals and caffeinated drinks or supplements at least 2 h before the sessions and to wear athletic clothing and neutral sports shoes.

**Table 2:** Trainees' characteristics (mean ± SD)

	Men (*n* = 4)	Women (*n* = 4)
Age	31.7 ± 6.3	29.7 ± 9.0
Years of experience in any workout regime	16.5 ± 5.5	18.0 ± 11.7
Years of experience in RT	9.0 ± 2.6	4.0 ± 2.8
Weight	82.5 ± 12.8	61.7 ± 17.2
Height	180.0 ± 6.6	163.2 ± 8.1
Average workouts per week	4.0 ± 0.8	2.5 ± 0.6

At the beginning of each session, the trainees completed a general warmup consisting of structured dynamic stretching and calisthenics and a five-min individualized self-selected warmup. They then completed an exercise-specific warmup consisting of a gradual increase of the lifted loads toward an estimated 1RM, or the target load of the session before each exercise (see Emanuel et al. [13] for a detailed account of the warmup). Trainees were instructed to perform the concentric portion of each repetition as fast as possible while attempting to maintain a controlled ~ 2 s descend until lightly touching the box below them (individually set to achieve a knee angle of approximately 60–65 degrees) in the squat, or until fully extending their elbows in the biceps-curl exercise, after which they immediately began the concentric portion. In the two last sessions, eight minutes of rest were provided between sets and exercises.

### Video Recordings

**Fig. 1:**
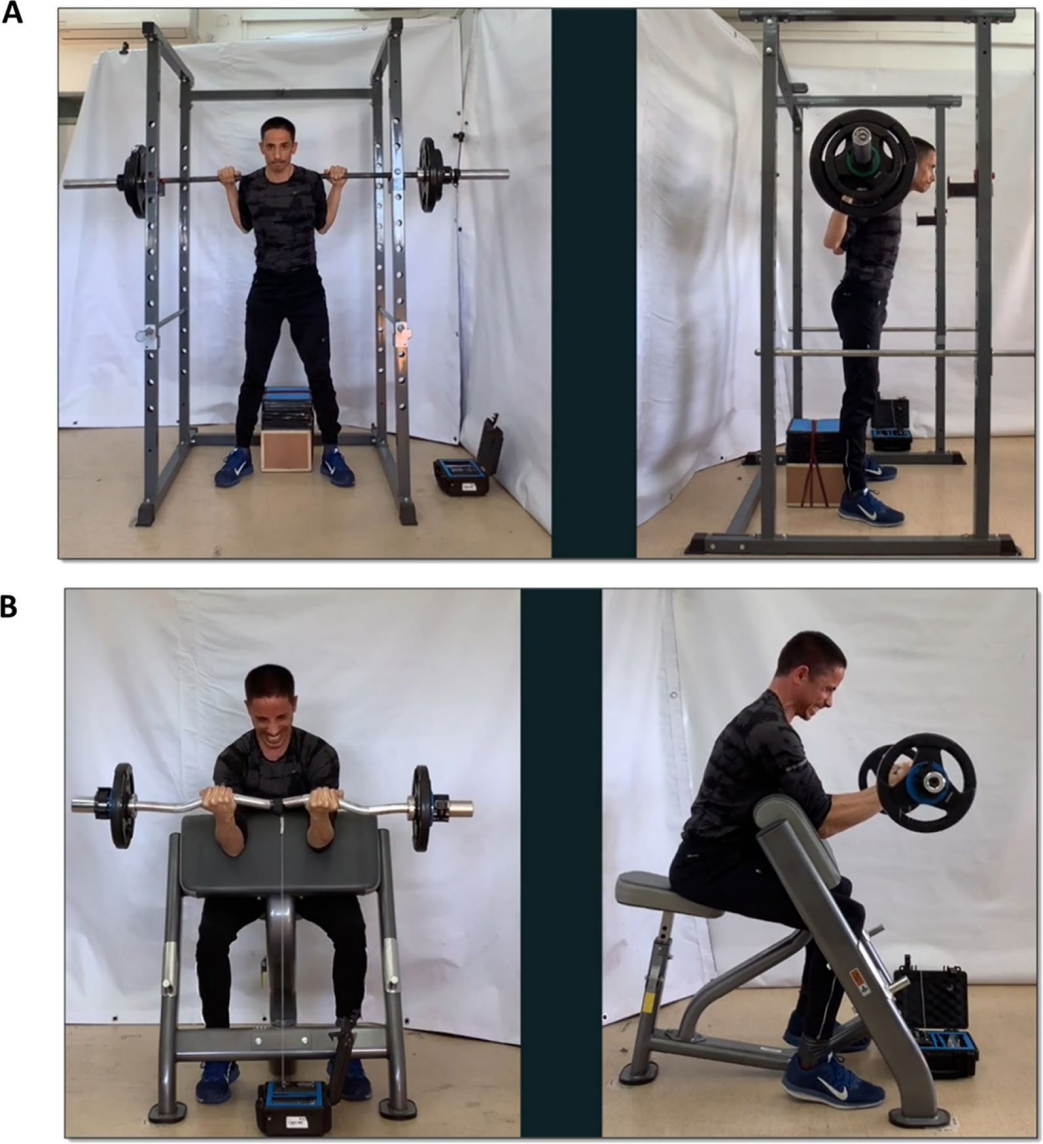
Video recording. An example of a snapshot from a video the coaches watched and rated at three time-points during each set

Video recordings were taken via two Apple iPad Airs (Apple, CA, USA), fixated using a designated tripod at the same angles and heights. All videos were recorded with an image size of 720 by 1280, with an added side illumination ~ 1 m to the left of the front camera. We recorded the trainees from their front and from a 90 degrees angle to their left (Fig. 1). These angles were selected as they provided relevant information on the form of the two exercises. For example, a front view enables the detection of facial expressions and asymmetry between the limbs, while a side view enables the detection of movement in the upper and lower back. The recording setup was fixed per trainee across the two last sessions via tape marks on the floor and was set at a distance of 1.5–2 m and 2.5–3 m for the side and front views, respectively. The videos were then imported into Final Cut Pro HD (Version 4.5, Apple, CA, USA), where they were synchronized, edited further, and combined into a single.MOV file. All trainees signed informed consent forms approving their videos being published and distributed to participants as part of this study as approved by the Institution's Ethics Committee.

### Statistical Analysis

Two outcome measures were defined: the coaches' raw error and the absolute error (raw error = predicted repetitions − actual repetitions; absolute error =|predicted repetitions − actual repetitions|). The two measures are shown to convey both the direction of the prediction error and its magnitude. These were predicted during 33%, 66%, and 90% of the repetitions performed by the trainees before reaching task-failure. For example, assuming a trainee completed 15 repetitions of a given set, a coach was asked to predict the RIR at 33% of the set—i.e., after 5 repetitions. Consequently, the trainee had ten repetitions left before reaching task-failure. If the coach predicted that the trainee had eight repetitions left, then she made a raw error of − 2 repetitions and an absolute error of 2 repetitions.

We fitted a linear mixed model with the following predictors: %1RM (70% or 80%), set number (first or second), coach’s experience (years as a coach), coach’s gender (male or female), exercise (squat or biceps-curl), prediction time-point (33%, 66%, or 90% of the repetitions performed), trainee’s gender (male or female), and the interaction between the gender of the coach and the trainee. We added random intercepts to account for dependencies of each coach, as they provided repeated ratings per video, and of each trainee, as their videos were rated by several coaches. The final regression model, comprising the same independent variables, has been fitted to both raw error and absolute error, where coaches and trainees are denoted by *p* and *m,*, respectively:

$$\begin{aligned} {\text{absolute}}\;{\text{error}}_{{{\text{pm}}}} ({\text{raw}}\;{\text{error}}_{{{\text{pm}}}} ) & = ~\beta _{{0\;{\text{pm}}}} + \beta _{{33\% \;{\text{time}}\;{\text{point}}}} \times 33\% \;{\text{time}}\;{\text{point}}_{{{\text{pm}}}} \\ & \quad + \beta _{{90\% \;{\text{time}}\;{\text{point}}}} \times 90\% \;{\text{time}}\;{\text{point}}_{{{\text{pm}}}} + \beta _{{{\text{load}}}} \\ & \quad \times ~80\% ~1{\text{RM}}\;{\text{load}}_{{{\text{pm}}}} + \beta _{{{\text{squat}}\;{\text{exercise}}}} \times {\text{squat}}\;{\text{exercise}}_{{{\text{pm}}}} \\ & \quad + \beta _{{{\text{years}}\;{\text{of}}\;{\text{RT}}\;{\text{experience}}}} \times ~{\text{years}}\;{\text{of}}\;{\text{RT}}\;{\text{experience}}_{{\text{p}}} \\ & \quad + \beta _{{{\text{trainee}}\;{\text{gender}}}} \times {\text{male}}\;{\text{trainee}}_{{\text{m}}} + \beta _{{{\text{coach}}\;{\text{gender}}}} \times {\text{male}}\;{\text{coach}}_{{\text{p}}} \\ & \quad + \beta _{{{\text{coach}}\;{\text{trainee}}\;{\text{gender}}\;{\text{interaction}}}} \times {\text{trainee}}\;{\text{gender}} \times {\text{coach}}\;{\text{gender}}_{{{\text{pm}}}} \\ & \quad + \beta _{{{\text{reps}}}} \times {\text{number}}\;{\text{of}}\;{\text{repetitions}}\;{\text{performed}}_{{\text{m}}} + \beta _{{{\text{set}}}} \times {\text{set}}~2_{{\text{m}}} \\ \end{aligned}$$

where the intercept is comprised of the overall intercept and the coach ($P_{{0{\text{p}}}}$) and trainee ($M_{{0{\text{m}}}}$) random intercepts: $\beta_{{0\;{\text{pm}}}} = {\upgamma }_{00} + P_{{0\;{\text{p}}}} + M_{{0\;{\text{m}}}}$.

Upon inspection of the regression model residuals, heterogeneity of the variance was detected for absolute error. Hence, linear mixed models with robust estimates of the standard errors were used in the model where the absolute error was the dependent variable. The conditional and marginal *R*^2^ for the mixed regression models were calculated to quantify the explained variance.

Significance was set at *p* < 0.05. Statistical analyses and figures were carried out with R (version 4.0.2) using the following packages: robustlmm, ggplot2, and Performance. All data collected are available at https://osf.io/fgycv/.

## Results

**Fig. 2:**
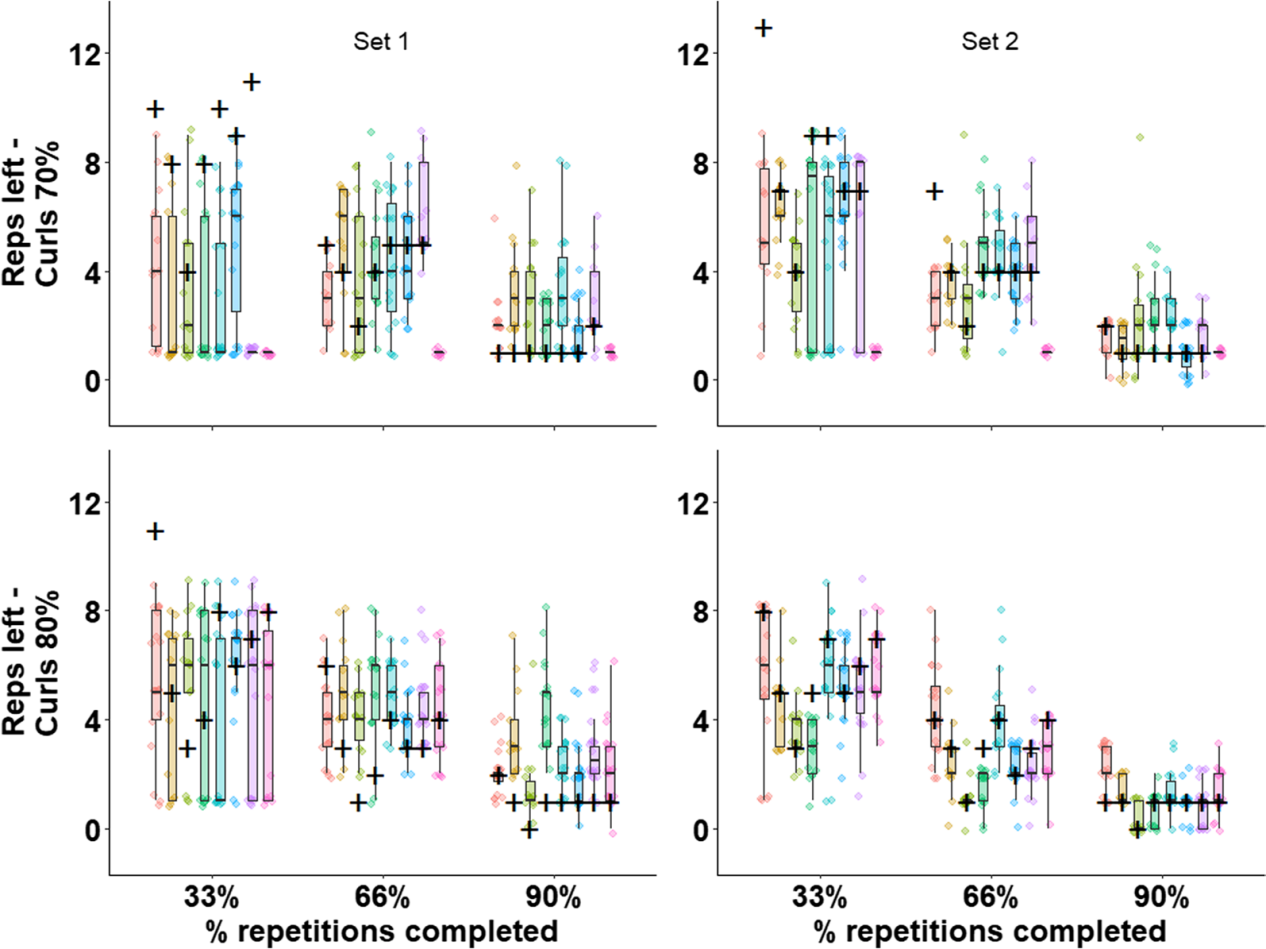
The predicted and actual repetitions to task-failure in the squat stratified by set, load, and trainee. The actual number of repetitions left for each trainee for a given time-point is represented by a cross. For each trainee, the predicated number of repetitions given by each coach is represented by a single-colored dot. The distribution of coaches’ predictions for each trainee is provided by a consistently ordered and colored boxplot. For example, at the top left panel, the black horizontal line in the leftmost boxplot (at the 33% mark, in red) indicates that the coaches predicted a median of four repetitions left for this trainee at the first prediction point, at the first set. However, the actual number of repetitions left for this trainee, under that condition, was 10 (black cross). Moving to the following prediction point (66%), for the same trainee (red), under the same condition, the median predicted repetitions left was three, whereas the actual number of repetitions left was five

**Fig. 3:**
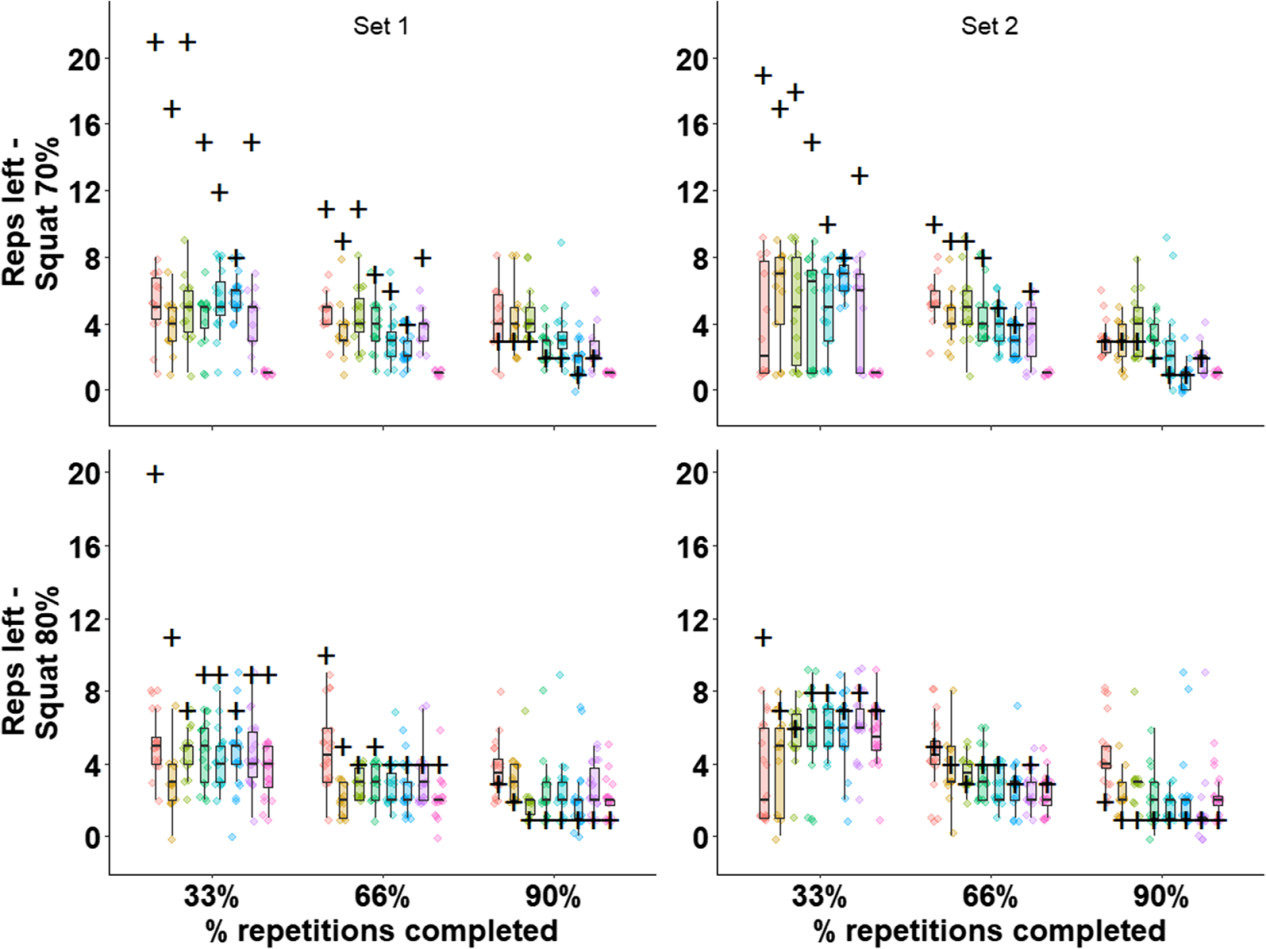
The predicted and actual repetitions to task-failure in the biceps-curl stratified by set, load, and trainee. Analogously to Fig. 2, the actual number of repetitions left for each trainee for a given time-point is represented by a cross. For each trainee, the predicted number of repetitions given by each coach is represented by a single-colored dot. The distribution of coaches’ predictions for each trainee is provided by a consistently ordered and colored boxplot

We plotted the actual and predicted repetitions left for each trainee at each time-point, exercise set, and load in Figs. 2 and 3. The marginal average absolute errors across the entire data were 4.8, 2.0, and 1.2 for the 33%, 66%, and 90% time-points, whereas the overall average number of repetitions completed by the trainees was 13.9 (see Table 3 for descriptive data). The marginal average raw error across the entire data was − 4.4, − 1.0, and 1.0 (where a minus sign indicates underprediction) for the 33%, 66%, and 90% time-points, respectively.

**Table 3:** Descriptive statistics (mean ± SD) of coaches’ predicted repetitions and actual repetitions performed by the trainees

	Predicted 33%	Actual 33%	Predicted 66%	Actual 66%	Predicted 90%	Actual 90%
Squat set-1 70%	4.8 ± 1.8	14.8 ± 4.6	3.6 ± 1.5	7.6 ± 2.5	3.2 ± 1.7	2.2 ± 0.7
Squat set-2 70%	5.2 ± 2.8	13.6 ± 4.0	4.0 ± 1.6	6.9 ± 2.2	2.4 ± 1.7	2.0 ± 0.8
Squat set-1 80%	4.3 ± 1.7	10.2 ± 4.0	2.9 ± 1.5	5.0 ± 2.0	2.6 ± 1.5	1.3 ± 0.7
Squat set-2 80%	5.3 ± 2.2	7.8 ± 1.4	3.1 ± 1.5	3.7 ± 0.6	2.4 ± 1.8	1.1 ± 0.3
Curl set-1 70%	3.4 ± 2.9	8.6 ± 2.0	4.4 ± 2.1	4.3 ± 1.0	2.5 ± 1.6	1.1 ± 0.3
Curl set-2 70%	5.9 ± 2.6	7.9 ± 2.4	3.9 ± 1.6	4.1 ± 1.9	1.7 ± 1.2	1.1 ± 0.3
Curl set-1 80%	5.0 ± 2.8	6.7 ± 2.3	4.4 ± 1.5	3.7 ± 1.3	2.6 ± 1.5	1.0 ± 0.4
Curl set-2 80%	4.9 ± 1.9	5.9 ± 1.42	2.6 ± 1.5	3.0 ± 0.9	1.1 ± 0.8	0.9 ± 0.3

To analyze the coaches’ prediction patterns, we fitted a regression model to coaches’ raw and absolute prediction errors. Due to a technical error in the online platform, we were unable to obtain 106 data points out of the total of 259 for experience and gender variables. Thus, we also ran the same robust linear mixed models without these variables. Both models resulted in similar marginal and conditional *R*^2^ values (Table 4). The results of the statistical models including all *n* = 259 participants, and of the statistical models including only *n* = 153 coaches with complete covariate data, are reported in Tables 4 and 5. The statistical models yielded very similar results for the overlapping covariates for both prediction errors. This is aligned with the missing data being attributable to software issues and is also supported by the similarities of the non-missing covariates between the trainees (see Additional file 1: Table S1). Hence, we elaborate on the statistical model results for the *n* = 153 coaches.

**Table 4:** Mixed regression models predicting absolute prediction error (repetitions completed—centered)

	*N* = 259; marginal *R*^2^ = 0.37; conditional *R*^2^ = 0.48	*N* = 153; marginal *R*^2^ = 0.35; conditional *R*^2^ = 0.46
Variable	Estimate [95% CI], *p* value	Estimate [95% CI], *p* value
Intercept	4.53 [3.57, 5.46] < 0.001	4.52 [3.67, 5.38] < 0.001
66% versus 33% failure proximity	− 1.60 [− 1.82, − 1.38] < 0.001	− 1.49 [− 1.75, − 1.22] < 0.001
90% versus 33% failure proximity	− 2.07 [− 2.36, − 1.78] < 0.001	− 2.05 [− 2.40, − 1.69] < 0.001
Set 2 versus set 1	− 1.21 [− 1.36, − 1.07] < 0.001	− 1.20 [− 1.38, − 1.02] < 0.001
Squat versus biceps-curls	2.23 [1.96, 2.50] < 0.001	1.43 [1.13, 1.74] < 0.001
80% versus 70%1RM	− 1.10 [− 2.22, 0.013] 0.052	− 1.17 [− 2.16, − 0.19] 0.022
Repetitions completed (centered)	− 0.11 [− 0.14, − 0.08] < 0.001	− 0.07 [− 0.11, − 0.04] < 0.001
Years of training experience (centered)	–	− 0.020 [− 0.039, − 0.0007] 0.042
Male versus female trainee	− 0.59 [− 1.70, 0.51] 0.266	− 0.47 [− 1.46, 0.52] 0.324
Male versus female coach	–	0.03 [− 0.23, 0.30] 0.810
Male versus female coach X male versus female trainee	–	0.03 [− 0.35, 0.43] 0.843
Squat versus biceps-curls X 80% versus 70%1RM	− 1.37 [− 1.67, − 1.06] < 0.001	− 0.80 [− 1.15, − 0.45] < 0.001

*CI* confidence interval

**Table 5:** Mixed regression models predicting raw prediction error (repetitions completed—centered)

	*N* = 259; Marginal *R*^2^ = 0.44; Conditional *R*^2^ = 0.54	*N* = 153; Marginal *R*^2^ = 0.42; Conditional *R*^2^ = 0.52
Variable	Estimate [95% CI], *p* value	Estimate [95% CI], *p* value
Intercept	− 4.26 [− 5.54, − 2.99] < 0.001	− 4.40 [− 5.67, − 3.12] < 0.001
66% versus 33% failure proximity	3.02 [2.71, 3.32] < 0.001	2.88 [2.50, 3.26] < 0.001
90% versus 33% failure proximity	4.81 [4.41, 5.21] < 0.001	4.47 [3.97, 4.98] < 0.001
Set 2 versus set 1	0.64 [0.44, 0.84] < 0.001	0.59 [0.34, 0.84] < 0.001
Squat versus biceps-curls	− 3.48 [− 3.85, − 3.11] < 0.001	− 2.75 [− 3.19, − 2.32] < 0.001
80% versus 70%1RM	1.34 [− 0.15, 2.83] 0.074	1.56 [0.09, 3.03] 0.038
Repetitions completed (centered)	0.08 [0.04, 0.12] < 0.001	0.06 [0.01, 0.11] 0.0183
Years of training experience (centered)	–	0.002 [− 0.025, 0.029] 0.890
Male versus female trainee	0.59 [− 0.88, 2.07] 0.398	0.58 [− 0.90, 2.07] 0.410
Male versus female coach	–	0.01 [− 0.36, 0.40] 0.927
Male versus female coach X male versus female trainee	–	0.02 [− 0.53, 0.58] 0.928
Squat versus biceps-curls X 80% versus 70%1RM	1.84 [1.43, 2.26] < 0.001	1.29 [0.79, 1.80] < 0.001

*CI* confidence interval

The model’s intercept was estimated at 4.52 (95% CI [3.67, 5.38]). In our formulation, the intercept represents the estimated average absolute error, at the average values of the above variables, for the biceps-curl exercise, at the first set, at 70% 1RM at the 33% time-point. Progressing to the 66% and the 90% time-points reduced the error further by − 1.49 (95% CI [− 1.75, − 1.22]) and − 2.05 (95% CI [− 2.40, − 1.69]), respectively. When participants observed the second set of each exercise, the absolute error was further reduced by − 1.20 (95% CI [− 1.38, − 1.02]). A significant interaction term between the exercise and load warrants a slightly different interpretation of these two variables: Changing the exercise from biceps-curl at 70% 1RM to squats at 70% 1RM increased the error by 1.43 (95% CI [1.13, 1.74]); changing the load in biceps-curl from 70% 1RM to 80% 1RM reduced the error by − 1.17 (95% CI [− 2.16, − 0.19]); and changing the load in the squat from 70% 1RM to 80% 1RM reduced the error by − 0.80 (95% CI [− 1.15, − 0.45]). The model also revealed that experience has a negligible but significant reduction of the absolute error by 0.02 per year of experience (95% CI [− 0.039, − 0.0007]). Finally, the number of repetitions completed before a time-point reduced the absolute error by − 0.07 (95% CI [− 0.11, − 0.04]) per repetition performed.

We further modeled the raw prediction error to infer over- and underpredictions of the coaches’ predictions, adjusted for other variables. Using the same intercept definition as in the absolute error regression model, we found that coaches underpredicted the number of repetitions remaining to failure at the 33% time-point by − 4.40 (95% CI [− 5.67, − 3.12]). The initial underprediction decreased by 2.88 (95% CI [2.50, 3.26]) repetitions at the 66% time-point. Moreover, the initial underprediction changed to a slight overprediction at the 90% time-point, for which an additional raw error of 4.47 (95% CI [2.50, 4.98]) was estimated by the regression model.

## Discussion

In this study, we analyzed coaches’ prediction of the RIR of RT trained trainees who completed two sets of two different exercises using two different loads. We found that the following variables improved coaches’ absolute prediction error: later predictions during sets, biceps-curl (compared to squat), using heavier loads, the second set, more completed repetitions at the time of predictions, and greater coaching experience, although the latter had a negligible effect. Furthermore, analysis of the raw error showed that coaches tended to underpredict the RIR in the first and second prediction time-points but reverted to overprediction in the final prediction point.

The higher prediction accuracy observed in the biceps-curl compared to the squat can stem from several reasons. First, the dynamic portion of the biceps-curl occurs in the elbow joint, whereas in the squat it occurs in the ankle, knee, and hip joints. Accordingly, it is possible that coaches directed their attention to a smaller area where movement occurred and extracted information that led to better predictions. Second, the extent to which trainees can modify exercise execution and thus compensate for muscular fatigue differs between the two exercises. In the biceps-curl, the arms are fixated to the preacher curl device, making it difficult to modify exercise execution. Hence, when fatigue of the elbow flexors accumulates, trainees are restricted in their ability to involve other muscle groups to assist in completing further repetitions. Conversely, when squatting, trainees are less restricted in their movements and can thus alter exercise execution and increase the involvement of different muscle groups [16, 17], for example, to compensate for quadriceps fatigue, trainees may implement greater hip flexion, which leads to increased involvement of the hamstring and gluteal muscles [18, 19]. We therefore speculate that exercises that offer fewer opportunities for exercise modifications lead to better RIR predictions.

The better RIR prediction in the second compared to the first set suggests a learning effect. Coaches were likely able to collect information about the trainees’ abilities in the first set, leading to improved predictions in the second set. Note that before this study, the coaches did not observe the trainees perform the exercises and received minimal information about their abilities. It is thus likely that predictions would have further improved if the coaches had received greater exposure to the trainees performing the exercises. Surprisingly, the effect of coaching experience on prediction accuracy was negligible, although statistically significant. This result aligns with a meta-analysis inspecting trainees’ prediction of the RIR, in which trainees’ RT experience was negligibly associated with accurate predictions of the RIR [10]. Collectively, the immediate improvements in predictions over sets, coupled with the negligible effects of coaching experience, suggest that prediction accuracy of RIR does not generalize well across trainees. Instead, this ability likely depends on coaches’ specific knowledge of their trainees’ unique abilities.

The prediction accuracy was higher when provided at later time-points or after more repetitions were completed. This may stem from several reasons: First, coaches inferred that predictions at later time-points, or after more repetitions, are closer to task-failure. Second, coaches improved at identifying signs of fatigue exhibited by the trainees. Third, and complementary to the second reason, the trainees exhibited greater signs of fatigue, which is associated with alteration in movement execution [20, 21]. The latter reason can also explain the improvement in prediction under heavier loads, where trainees may have exhibited greater signs of fatigue at each repetition. Unfortunately, the current study design cannot disentangle the effects of these proposed reasons.

With respect to the raw prediction error, coaches changed their prediction patterns over successive prediction time-points. Coaches significantly underpredicted the RIR in the first time-point. This bias remained during the second time-point although its magnitude decreased. By the third time-point, coaches were the most accurate and even slightly overpredicted the RIR. While we are uncertain why coaches tend to underpredict the RIR at the beginning of sets, awareness of this bias may be of practical importance. For example, coaches can deliberately add repetitions to their RIR predictions at the early stages of a trainee’s set.

This study has several limitations worthy of discussion. First, coaches typically observe trainees complete exercises in person rather than viewing them on a screen. While we provided coaches with two viewing angles of the exercising trainees, in-person coaching allows for varying viewing angles and other nuanced information that is absent from a screen. Future research could assess coaches’ predictions in a gym environment rather than videos. Second, coaches observed the exercises and sets in a fixed order: squats preceded the biceps-curls, and the first set preceded the second set. The structure of this study design may have led to an order effect. Future research can overcome some of these limitations by presenting different segments of the videos in a randomized order. For example, by first presenting the set that was performed second or presenting the last portion of a set before the first one. This will allow testing the proficiency of coaches in predicting the RIR in isolation from the other parts of the video viewed before. Third, the trainees’ average number of repetitions in the first set of the squats using 70%1RM was 22. This value is considerably higher than the 8–12 repetition range that might be expected at 70%1RM according to some textbooks [22, 23]. Coaches may have anticipated such a repetition range, which could have led to lower RIR estimations. However, we note that several studies report repetitions values that are consistent with those observed in the present study, suggesting that this repetitions range can be representative of what occurs in everyday training environments [13, 14, 24–26].

## Conclusions

We have shown that the accuracy of coaches’ predictions of RIR depends on several variables. Mainly, predictions improve when coaches provide them in later stages of a set, when using heavier loads, in later sets, and with the biceps-curl. Conversely, coaching experience played a trivial role in improving prediction accuracy. These results are mostly aligned with a recent meta-analysis inspecting trainees’ RIR predictions [10]. Prediction accuracy improved when trainees provided their predictions closer to task-failure, when using heavier loads, in later sets, and was independent of trainees RT experience. In the present study, coaches also tended to underpredict the RIR in the first prediction, but this effect shrunk and eventually turned to an overprediction by the final prediction. Practically, these results suggest that coaches’ ability to predict task-failure is less accurate at the beginning of a set but tends to improve as sets progress, with consecutive sets, and for sets composed of heavier loads.

## Supplementary Information

**Additional file 1.** Comparisons between demographics of complete and missing data.

## Data Availability

The dataset supporting the conclusions of this article is available in the Open Science Framework repository, https://osf.io/fgycv/.

## Abbreviations

RIR: Repetitions in reserve
RM: Repetition maximum
RT: Resistance training
CI: Confidence interval
