# Supplementary material for: Seeing Effort: Assessing Coaches' Prediction of the Number of Repetitions in Reserve Before Task-Failure
Source: Sports Med Open. 2022 Oct 22;8:132. doi: 10.1186/s40798-022-00526-8 (PMC9588140; doi:10.1186/s40798-022-00526-8)
Supplement: Supplementary file 1 — Additional file 1. Comparisons between demographics of complete and missing data. [file 40798_2022_526_MOESM1_ESM.docx]

**Table 1.**

| Comparisons between demographics of complete and missing data in Table 1 | | | |
| --- | --- | --- | --- |
|  | Complete Data | Missing Data |  |
| n | 153 | 106 | standardized mean difference |
| Age (mean (SD)) | 30.04 (7.39) | 28.53 (8.96) | 0.18 |
| Weight (mean (SD)) | 74.26 (12.63) | 74.64 (12.67) | 0.03 |
| Height (mean (SD)) | 173.65 (9.04) | 173.84 (10.08) | 0.20 |
| Average workouts per week (mean (SD)) | 4.59 (3.19) | 5.76 (4.04) | 0.32 |
